# The Investigation of Quality of Life in 87 Chinese Patients with Disorders of Sex Development

**DOI:** 10.1155/2015/342420

**Published:** 2015-05-17

**Authors:** Chunqing Wang, Qinjie Tian

**Affiliations:** Department of Obstetrics and Gynecology, Peking Union Medical College Hospital, Chinese Academy of Medical Sciences, Peking Union Medical College, No. 1 Shuaifuyuan, Wangfujing Street, Dongcheng District, Beijing 100730, China

## Abstract

*Objective*. In the process of care for disorders of sex development (DSD), clinical decisions should focus on the long-term quality of life (QOL). We sought to investigate the QOL of patients with DSD in China.* Design*. Case-control study was carried out.* Patients.* 90 patients of DSD participated in the study. Finally, 87 patients were analyzed including Turner's syndrome (23), Noonan syndrome (2), androgen insensitivity syndrome (22), testicular regression syndrome (2), congenital adrenal hyperplasia (16), and pure gonadal dysgenesis (22).* Measurements*. The WHOQOL-BREF questionnaire was chosen for the present investigation. Four domain scores were analyzed independently including physical, psychological, and social relationship and environmental domains.* Results*. The average age of the DSD group is 22.34 ± 4.97 years, and only 13.79% patients ever had sexual life. The scores of psychological and environmental domains were lower than that of the physical and social relationship domains, but the difference was not significant (*P* > 0.05). Compared with the Chinese urban population, the QOL scores of DSD patients in China were not significantly lower.* Conclusions*. With proper treatment, including the follow-up and psychological support, the QOL of DSD patients cannot be significantly reduced. For DSD patients, more attention should be paid to the potential psychological and sexual problems.

## 1. Introduction

Disorders of sex development (DSD) were an umbrella name for a group of congenital developmental disorders in which the chromosomal, gonadal, or anatomical sex is atypical [1]. According to European Society for Paediatric Endocrinology (ESPE), DSD can be classified into three kinds: (1) sex chromosome DSD, such as Turner's syndrome (TS, 45, X, or another mosaic); (2) 46, XY DSD, such as androgen insensitivity syndrome (AIS) and testicular regression syndrome (TRS); and (3) 46, XX DSD, such as 46, XX pure gonadal dysgenesis (PGD), and congenital adrenal hyperplasia (CAH) [1]. The epidemiological data of DSD is limited and its incidence varies between races and ethnic groups. The worldwide average is 1 per 5,500, whereas among newborns in Germany it is 2 per 10,000 [2–4]. The typical manifestations of DSD patients include ambiguous genitalia after birth or puberty, absence of secondary sexual characteristics after puberty, primary amenorrhea, short stature, or substantial height. The clinical diagnosis and management of DSD are difficult and complex because of the various aetiology and diverse manifestation, which include four main issues: aetiological diagnosis, gender assignment, indication for and timing of gonadal and genital surgery, and a careful explanation of the nature of the condition and its consequences to the patients and their parents [5–7]. The effects of DSD management lie not only on its correct diagnosis but also on the concern of results of long-term treatment and quality of life (QOL). Importantly, a multidisciplinary approach is preferred and should include a neonatologist, gynaecologist, endocrinologist, psychiatrist, geneticist, surgeon, and a social worker [8]. Optimal treatment is patient- and family-centred and delivered by interdisciplinary teams [9]. In particular, the pressing question of every clinician caring for DSD patients remained, such as the relationship between gender assignment, future QOL, and adaption [10]. Although there have been some researches on QOL of DSD patients [11], larger studies of subjects with different DSD are still needed. We assumed that the DSD patients' QOL to be impaired because of the discrepancy between social gender and chromosomal sex, lack or excess of sex hormone, long-term need of sex hormones and other medications, health risks such as tumor, the negative influence on marriage and reproduction, and the related psychological pressure. Our observation may provide valuable proof or new insight for a better DSD care.

## 2. Patients and Methods

### 2.1. Patients

90 patients of DSD who came to Peking Union Medical College Hospital (PUMCH) from different regions of China participated in the study from September 2012 to December 2013. The inclusion criteria were as follows. (1) All patients had a definite diagnosis after a comprehensive checkup including characteristics of clinical manifestation, laboratory tests of chromosomal karyotype and hormone assays, or results of pathology after surgery. (2) All patients were over 13 years old. (3) The DSD patients were all treated for more than half a year. (4) All patients signed informed consent. The exclusion criteria included (1) lack of definite diagnosis, (2) congenital reproductive tract dysplasia, such as the congenital absence of uterus and vagina, and (3) patients who had severe mental disorders and could not understand and answer the questionnaire properly. The contents of data collected included name, gender, educational level, employment status, the specific diagnosis, and the scores of the WHOQOL-BREF questionnaire. The participants were divided into four groups: TS, CAH, AIS, and PGD.

### 2.2. Methods

The WHOQOL-BREF questionnaire was chosen for the present investigation for several reasons. First, it is one of the most commonly used generic QOL questionnaires and has an official Chinese version approved by the WHOQOL Group. Second, it is convenient for clinical trials because of its short length. It contains 26 items, including two separately analytical items: item 1 and item 2. The other 24 items can produce four domain scores: physical, psychological, social relationship, and environmental domains. The mean score of items within each domain is equivalent to the domain score which is scaled in a positive direction. They can also be transformed to a 0–100 scale using the following formula: transformed score = (Score − 4) × 4 × (100/16) [12]. The DSD patients were told that the questionnaire was about the situations in the past four weeks and should be filled out according to their first impression and then finished the self-reported QOL independently. If more than 20% of data are missing from an assessment, the questionnaire will be discarded. If up to two items are missing, the mean of other items in the domain can be substituted. If more than two items are missing from the domain, the domain score should not be calculated (with the exception of social relationship domain, where the domain should only be calculated if no more than 1 item is missing).

### 2.3. Data Analysis and Statistical Methods

SPSS 16.0 software was used to analyze the data collected. Descriptive statistics of percentages, mean, and standard deviation were used to summarize the basic data and QOL scores. For the comparison between different DSD groups, Kolmogorov-Smirnov test was first used to detect the distribution of the data. Homogeneity of variances was done with the normal distribution data and one-way ANOVA was considered about the data with constant variances. For the *t*-test, a *P* value less than 0.05 was considered statistically significant.

## 3. Results

Among the 90 patients, two cases did not return the questionnaire and one patient could not answer it because of severe depression, leaving 87 suitable for inclusion. The patients were 13–38 years old with a mean age of 22.34 ± 4.97 years. The social gender of all the patients was female while the chromosomal karyotype differed: 21 (24.1%) were 46, XX, 43 (49.4%) were 46, XY, and 23 (26.4%) were either 45, X or another mosaic. The diagnosis of these DSD participants includes TS (23), Noonan syndrome (2), AIS (22), TRS (2), CAH (16), and PGD (22). Twelve patients (13.79%) of 18–34 years old ever had sexual activity while the rest 75 (86.21%) did not. Noonan syndrome has similar clinical manifestation with TS [13] and there are only two cases with Noonan syndrome in this study, so they were assigned into category of TS. Similarly, TRS patients have primary amenorrhea and no uterus with a 46, XY karyotype as patients of AIS [14], and they were arranged into AIS group for analysis because of small numbers. All characteristics of 87 patients were shown in Table 1.

The scores of the physical, psychological, social relationship and environmental domains were calculated, respectively, and transformed into 1–100 scale (Table 2). The scores of psychological and environmental domains are lower than that of the physical and social relationship domains, but the difference is not significant (*P* > 0.05). The scores of each domain were analyzed using Kolmogorov-Smirnov test and one-way ANOVA test between different groups. The data was normally distributed and the result of the ANOVA test demonstrated no difference (Table 3).

As was shown in Table 4, there was no significant difference between DSD patients and general population in Guangzhou, the capital city of Guangdong Province in China, reported by Xia et al. (as shown in Table 5) [15], about the scores of physical, psychological, and social relationship domains. Patients of chronic illnesses, such as hypertension, diabetes, and chronic rhinitis, scored less in physical and social relationship and environmental domains than DSD patients and the difference was significant (*P* < 0.01).

## 4. Discussion

QOL is defined as “an individual's perception of their position in life in the context of the culture and value systems in which they live and in relation to their goals, expectations, standards, and needs” according to the World Health Organization [12]. QOL is a comprehensive concept which encompasses the connection among personal, physical health, psychological status, independent ability, social relationship, personal beliefs, and environment. WHOQOL-BREF has been widely used in medical field since 1996 and can make it convenient to understand a disorder and develop treatment methods [12]. The QOL results of the 87 DSD patients in our study showed that the score of the psychological domain is lower than that of the physical and social relationship domains without significance (*P* > 0.05). This is in accordance with past report using different questionnaire [16]. Being potentially more severe than in other congenital conditions, the DSD patients' QOL is dependent on the extent of healthcare provider's attending to psychosocial aspects [17]. So the new ESPE DSD guidelines acknowledge the significance of psychosocial care and the involvement of mental health expertise except medical-surgical treatment.

CAH females had a poorer QOL compared to other DSDs in a case-control study [18]. But our study showed no significant difference among the four different kinds of DSD (*P* > 0.05). The possible reasons include different patient groups, questionnaires, and patients' races. The previous study divided the 70 Danish DSD patients into CAIS, virilized females, CAH, and gonadal dysgenesis (GD), using Quality of Life-Assessment of Growth Hormone Deficiency in Adults' (QoL-AGHDA) questionnaire. Interestingly, there were only 13.79% of patients of DSD having had sexual activities in our study. This may be related to the younger age of the patients and the traditional culture environment in China. In addition, insecurity and fear of rejection in the DSD patients may result in postponing intimate activities after realizing their health status [19]. Indeed evidence suggests that sexual problems exist more frequently in DSD patients than in the general population [20, 21]. Ros et al. [16] investigated the QOL and sexual function of 26 patients with TS aged 36.7 ± 8.4 years and discovered that only 13 (50%) cases reported sexual activity and had poorer arousal than the healthy control. In recent 30 years, clitoroplasty with preservation of neurovascular pedicles was applied for CAH patients with clitoromegaly in PUMCH, which is helpful for the reservation of organism and improving the quality of sexual life. According to our original plan, we also wanted to study the sexual situation of DSD patients, but the sample was too small and the data was not presented here. DSD patients are less likely to be actively involved in sexual activity. Clinicians should pay more attention to the sexual problems of DSD, providing not only the medical and sexual education but also the opportunity to discuss their problems with a mental health doctor [19].

Xia et al. [15] investigated the QOL of Chinese urban population and people with chronic illness defined as a medical condition diagnosed by a doctor at least six months before the study, for which either the symptom(s) persisted or relevant medical treatment continued. DSD is also a chronic disease. So the QOL scores of DSD patients were compared with the two “ensembles,” respectively. The DSD patients scored better than patients with other chronic illnesses mainly because the latter mainly included patients of hypertension, diabetes mellitus, and coronary heart disease and were older.

The QOL score of Chinese DSD patients was not significantly lower than that of the Chinese urban population. There are several possible reasons: (1) our patients can obtain good patient education and great psychological support from the doctors. We always try to explain the aetiology and prognosis in an easy to understand way and tell them the disorder itself is not the patients' or their parents' fault in order to relieve their stress and encourage them to “change what is changeable and accept what is not.” (2) Essential medical or surgical treatments were given to facilitate the development in accordance with their social gender, decrease osteoporosis or cardiovascular disease, and prevent tumorogenesis of the gonads. Proper treatment might have a greater influence on the patients than the genotype, as indicated in a recent study [22]. A longitudinal study on CAH also showed that daily-reduced hydrocortisone dose might be helpful for the improvement of QOL [23]. (3) To protect their personal privacy, we adopt patient-centred service concept. (4) We provide psychological support and counseling on marriage, sexual life, and fertility. For example, 46, XX CAH patients can have their own babies; PGD patients can marry and oocyte donation might be an option to have children. (5) Regular follow-up was carried out. This can guarantee that they obtain essential medical information, proper treatment, and psychological support. Warne et al.'s research also demonstrated this with different QOL questionnaire [24]. The patient with severe depression may prevent such a condition if she returned to see a doctor regularly.

Although there are some QOL studies about single CAH, TS, or AIS [25–32], the literature about QOL of different DSD patients together is very limited [18, 33, 34]. Our study has the greatest sample size in QOL study about DSD including subtypes. The QOL investigation of 50 DSD patients by Zhu et al. [33], including children aging 6–11 years and adolescents aged 12–17 years, showed that they had an impaired QOL after plastic surgery compared to control group. Two of the patients recruited in their study were diagnosed with ovotestis which is not included in our study. Among the 50 patients, 8 persons were raised as boys according to their social gender and 4 of them suffered psychological abnormality, including various degrees of depression, poor communication, hyperactivity, or discipline problems. Adults may have adapted better to their circumstances than children and adolescents, although this discrepancy may also be due to the different questionnaires used. However, no significant difference in the psychological problems was observed between girls with DSDs and girls in the control group, which is consistent with our study. The higher prevalence of satisfaction in girls may have contributed to their better accommodation with social environments. There are various results of QOL in DSD patients. Some scored better and some appeared severely impaired compared to reference groups [16, 27, 35]. QOL of DSD patients may be influenced by the support from their parents, doctors, and friends, the patients' personality and ability to accept their conditions, and the medical and surgical care [11].

There are several limitations of this study. (1) Our study may have a case selection bias. The data was collected in gynecological clinic in PUMCH, one of the best hospitals with specialist in DSD field in China, and all the participants were female. DSD may not be well recognized in some other districts and patients there may have worse QOL as in some other developing countries [36]. (2) This is a single center experience. The participants, most of whom were outpatients, were all definitely diagnosed, had received treatment more than half a year, and had the opportunity to be followed up. (3) We do not have their data of QOL at initial visit and cannot make a longitudinal comparison. This is the first QOL study of patients with DSD in China. Larger prospective studies may be needed for a better understanding of the DSD patients' QOL and curative effects.

In conclusion, the QOL of DSD patients can be hopefully unaffected after proper treatment, especially follow-up and psychological support. For DSD patients, more attention should be paid to the potential psychological and sexual problems, so we should emphasize the psychological and sexual education, treatment, and intervention during the multidisciplinary approach.

## Figures and Tables

**Table 1:** Basic characteristics of the 87 patients with DSD.

Variables	*n*	%
Age (years)		
Mean	22.34 (4.97)	
Range	13–38	
Social gender	Female	
Karyotype		
46, XX	21	24.1
46, XY	43	49.4
45, X or mosaic	23	26.4
Employment		
Employed	32	36.8
Unemployed	16	18.4
Schooling	39	44.8
Educational level		
Secondary	48	55.2
Postsecondary	39	44.8

**Table 2:** Scores of the four domains of QOL in each kind of DSD.

	Physical domain	Psychological domain	Social relationship	Environmental domain
TS	64.43 ± 12.81	60.67 ± 13.98	64.50 ± 18.09	61.38 ± 13.83
CAH	66.07 ± 13.17	58.85 ± 14.10	66.15 ± 15.05	58.20 ± 15.56
AIS	67.71 ± 15.24	64.93 ± 12.59	66.84 ± 16.91	63.02 ± 14.88
PGD	63.80 ± 15.60	59.28 ± 14.88	68.75 ± 19.99	58.24 ± 13.42

Total	65.48 ± 14.15	61.16 ± 13.84	66.52 ± 17.53	60.45 ± 14.25

**Table 3:** The ANOVA test results of the four domains between four different DSD diseases.

Statistic	Physical	Psychological	Social relationship	Environmental
*F* value	0.349	0.884	0.228	0.596
*P* value	0.790	0.453	0.877	0.619

**Table 4:** The *t*-test results of the comparison between the patients with DSD and patients with chronic diseases and Chinese urban people.

	Physical domain		Psychological domain		Social relationship		Environmental domain	
	CI^*^	CP^*※*^	CI	CP	CI	CP	CI	CP
*t*	3.129	1.308	1.516	0.107	2.869	1.459	5.180	5.599
*P*	0.002	0.194	0.133	0.915	0.005	0.148	0.000	0.000

^*^CI: chronic illness; ^*※*^CP: Chinese population.

**Table 5:** Scores of the four domains of QOL in CP and patients with CI reported by Xia et al. [15].

	Physical domain	Psychological domain	Social relationship	Environmental domain
CI	60.73 ± 11.94	58.91 ± 13.15	61.13 ± 11.79	52.54 ± 13.83
CP	67.46 ± 12.34	61.00 ± 14.15	63.78 ± 14.41	51.90 ± 14.72
